# Cross-cultural adaptation, reliability, and validity of the Turkish version of the Neck OutcOme Score

**DOI:** 10.3906/sag-1907-87

**Published:** 2019-12-16

**Authors:** Şeyda CANDENİZ, Seyit ÇITAKER, Batuhan BAKIRARAR

**Affiliations:** 1 Department of Physiotherapy, Vocational School, Ankara University, Ankara Turkey; 2 Department of Physiotherapy, Faculty of Health Sciences, Gazi University, Ankara Turkey; 3 Department of Biostatistics, Faculty of Medicine, Ankara University, Ankara Turkey

**Keywords:** Neck pain, validity, reliability, version, Neck OutcOme Score

## Abstract

**Background/aim:**

This study aims to determine the validity and reliability of the Turkish version of the Neck OutcOme Score (NOOS).

**Materials and methods:**

Two hundred eight patients suffering from nonspecific neck pain participated in the study. Test–retest reliability and internal consistency were assessed using intraclass correlation coefficients (2, 1) and Cronbach’s alpha, respectively. The dimensionality was investigated with the factor analysis. The construct validity was determined by testing whether the hypothesis of correlations between NOOS subscales, Short Form-36 subscales, and the Neck Disability Index were met using Spearman’s rank correlation coefficient. Ceiling/floor effects and measurement error were tested as well.

**Results:**

The intraclass correlation coefficient results varied between 0.721 and 0.844. Cronbach’s alpha values of the subscale were found to be between 0.847 and 0.916 in the internal consistency analysis. The factor analysis showed that the questionnaire has five factors. Floor/ceiling effects were considered not to be present.

**Conclusion:**

It was found that the Turkish version of the NOOS is valid and reliable.

## 1. Introduction

Neck pain is one of the three most reported complaints of the musculoskeletal system. In general, it was reported that prevalence is most common among around 50-year-old individuals, and it is higher among women than men [1]. It is estimated that between 22% and 70% of the population will experience some degree of neck pain in their lives [2,3].

Evaluating the level of neck pain is important in determining an individual’s quality of life, participation in everyday life, and limitations. The methods used for identifying the factors that cause these determined limitations and aggravate the pain include clinical examinations, psychological evaluations, and investigation of sociodemographic and economic factors. In addition to these evaluation parameters, functional scales are now used more commonly by clinicians and clinical researchers [4].

What is expected from scales is that they measure the status of individuals suffering from neck pain objectively and functionally and are sensitive to minor changes in individuals. The Copenhagen Neck Functional Disability Scale, Neck Pain and Disability Scale, and Neck Disability Index (NDI) are major questionnaires used for functional evaluation of neck pain in Turkey [5–7]. Among these questionnaires, the NDI is the most widely preferred [8,9]. Despite its common use in current methodological quality studies, the NDI has been criticized for its content validity, reliability, and dimensionality [9–11]. Moreover, a wide selection of patients, data saturation, and insufficient selection of samples and study populations when determining the content validity rendered the NDI inadequate [8]. The fact that neck pain triggers symptoms such as nausea, headache, and dizziness and that these symptoms have an impact on individuals’ participation in activities and the duration and quality of activities all increased the inadequacy of the NDI. Due to such shortcomings of the NDI, a need arose for a new scale that addresses neck pain symptoms extensively and evaluates the response of patients’ pain in their participation in different activities.

To assess the patients’ perception of their neck-related problems, the Neck OutcOme Score (NOOS) was developed by Juul et al. [12]. The NOOS is a 34-question questionnaire that investigates neck mobility, sleep disturbance, participation in everyday activities, quality of life, and neck symptoms. It involves five subscales: “mobility,” “symptoms,” “sleep disturbance,” “everyday activity and pain,” and “participation in everyday life.”

The questionnaire aims to measure a patient’s neck disability quantitatively according to the WHO’s International Classification of Functionality (ICF). The NOOS was developed within the framework of the ICF with a focus on body functions, structure, activity, and participation [13,14]. Hence, the NOOS meets the gaps in current scales. It is not yet available in any other language. Therefore, the aim of this study was to determine the validity and reliability of the Turkish version of the NOOS.

## 2. Materials and methods

This study was approved by Gazi University’s ethics committee (#77082166–604.01.04–70153). All participants gave informed consent, and all rights of the participants were protected. Two hundred eight patients between the ages of 18 and 65 years diagnosed with nonspecific neck pain were included in the study. Patients were excluded if they had a serious neurological disease, psychological distress, or alcohol or substance abuse.

According to the empirical approach, the sample size required for confirmatory factor analysis was determined as n = 200 or 5 times the number of items in the scale (n/items ≥5) [15]. If we calculate the number of questions (items = 34), we can say that a sample of 170 people is sufficient. In our study, we found it more appropriate to employ a sample size of n ≥ 200.

### 2.1. Cross-cultural adaptation

Translation and cultural adaptation were performed according to the method described by Beaton et al. [16]. Three forward translations from English into Turkish were performed by three independent bilingual translators (a medical health professional and two professionals without medical background and knowledge). All three versions were discussed and combined in a consensus meeting to provide a preliminary Turkish version. Two nonmedical translators and one medical translator translated the preliminary Turkish version back into English. The back-translation was discussed and compared with the English version. The preliminary version was tested with physically active patients suffering from neck problems for wording, understanding, and solid comprehension by experienced health professionals. First, this test procedure was performed involving 50 individuals of whom 15 were neck patients. The patients found it hard to understand questions M1 and M5, which asked about the mobility of neck extension and rotation. For this reason, items M1 and M5 were described more clearly. Next, comprehensiveness of the questionnaire was evaluated again in a pilot group of 35 sick subjects and 69 healthy subjects.

After approval by the original developers of the NOOS, final adjustments were made to obtain a final Turkish version of the NOOS. It was decided to abbreviated this as NOOS-Tr.

### 2.2. Validation study

The other part of the study included 208 patients between the ages of 18 and 65 years who suffered from nonspecific neck pain, were literate, and agreed to participate in the study. 

Data collection took place at the Gazi University Hospital and three private physiotherapy clinics between March and November 2017. The NDI, Short Form-36 (SF-36) [17], and NOOS-Tr questionnaires were applied to all patients during face-to-face interviews. For test–retest analysis, 71 of the patients completed the NOOS-Tr questionnaire one week later.

### 2.3. Statistical analysis

Analysis of the data was performed using SPSS 11.5 (SPSS Inc., Chicago, IL, USA). The descriptive statistics were mean ± standard deviation and median (minimum–maximum) for quantitative variables and the number of cases for qualitative variables (%). Test–retest and internal consistency analyses were conducted to determine reliability. Test–retest results were evaluated by the intraclass correlation coefficient (ICC) method. Cronbach’s alpha coefficient was used to evaluate the reliability of the questionnaire in terms of internal consistency. Cronbach’s alpha coefficient can be obtained in the absence of missing data in the dataset and a value of 0.70 is the minimum acceptable value. Multidimensionality of the items was analyzed by confirmatory factor analysis (CFA). Floor/ceiling effects and measurement error were tested as well. Significance was set at P < 0.05.

This study was guided by the COSMIN (Census-based Standards for the Selection of Health Measurement Instruments) recommendations to evaluate the methodological quality of measurement results and to verify that all important design features and statistical methods were reported clearly [18]. 

#### 2.3.1. Floor and ceiling effects

In this study, floor/ceiling effects were evaluated for NDI, SF-36, and NOOS subscales. Less than 15% of the participants who scored the lowest and the highest were determined to be acceptable [19,20]. 

#### 2.3.2. Internal consistency

Cronbach’s alpha values were calculated separately for each subscale to evaluate the internal consistency. Scores between 0.70 and 0.90 are considered sufficient for internal consistency [20–22].

#### 2.3.3. Test–retest reliability

In this study, test–retest reliability for the NOOS, NDI, and SF-36 was evaluated with ICC (2, 1). To perform the retest, it was decided that a one-week interval was a sufficient period during which clinical change would not occur.

#### 2.3.4. Error of measurement

The error of measurement was calculated using the standard error of measurement (SEM) as follows: SEM = SD × √(1 – Cronbach’s alpha). Here, SD refers to the standard deviation value of all participants’ baseline scores. 

To determine the minimal detectable change (MDC) with 90% confidence, the following formula was used: MDCind = 1.64 × SD × √(2 × (1 – r)) [23]. In the formulation, SD refers to the standard deviation value of all participants’ baseline scores, and r refers to the test–retest ICC value. If a change equivalent to or higher than the calculated MDCind value is observed, it can be said at a confidence level of 90% that the individual experienced an actual change. The MDCind value was also divided by √n to calculate the MDC (MDCgroup) values [19].

#### 2.3.5. Construct Validity

The study’s construct validity was evaluated by the predefined hypotheses developed based on the discussion of the current literature and clinical experience by the developers of the NOOS questionnaire [24]. The construct validity was determined by testing whether the hypothesis of correlations between NOOS subscales and the other instruments were met using Spearman’s rank correlation coefficient.

In positive correlation low, moderate, and high correlations were defined with 0.10–0.29, 0.30–0.49, and 0.50–1.0, respectively, for the correlation coefficient values, whereas ranges of –0.29 to –0.10, –0.49 to –0.30, and –1.0 to –0.5 were used to define low, moderate, and high correlations, respectively, for the negative correlation coefficient values [25]. It is inferred from the confirmation of 75% of the hypotheses that construct validity was achieved [19].

#### 2.3.6. Factor analysis

The purpose of CFA based on structural equation models is to determine whether predetermined relationship patterns between factors and items have been verified by data. With the help of the model’s goodness of fit statistics, it is decided whether the factors actually consist of these items. The most commonly used goodness of fit statistics are comparative fit index (CFI), Tucker–Lewis index (TLI), and root mean square error of approximation (RMSEA). CFI and TLI values greater than 0.90 indicate an acceptable fit and greater than 0.95 is considered to be a good fit. RMSEA values of less than 0.05 indicate a good fit, and less than 0.08 indicates an acceptable fit [26].

## 3. Results

Demographic characteristics of the patients are presented in Table 1. The participants, whose ages varied between 18 and 65 years, had a mean age of 35.88 ± 13.79, and their mean body mass index was found to be 25.02 ± 4.46.

**Table 1:** Demographic characteristics of patients with neck pain (n = 208).

Variables	n	%
Sex		
Female	145	69.7
Male	63	30.3
Education level		
Grade school	44	21.1
High school	36	17.3
Undergraduate	23	11.1
Graduate	85	40.9
Postgraduate	20	9.6
Smoking		
Yes	37	17.8
No	171	82.2
Comorbidities		
None	139	66.8
Cardiopulmonary disorders	20	9.6
Musculoskeletal disorders	22	10.6
Metabolic disorders	17	8.2
Neurological disorders	3	1.4
Internal medicine disorders	7	3.4
Surgery		
Yes	49	23.6
No	159	76.4
Duration of neck pain		
Acute	78	37.5
Chronic	130	62.5
The cause of neck pain		
Whiplash	54	26.0
Carrying heavy items	25	12.0
Trauma, fall	6	2.9
Hard work, activity	63	30.3
Others	60	28.8
Localization of neck pain		
Neck	53	25.5
Neck and shoulder	82	39.4
Neck, shoulder, and arms	73	35.1

### 3.1. Floor and ceiling effects

According to the initial values, no floor and ceiling effects were observed in the NOOS and NDI, and score distributions were found at acceptable levels in both (Table 2). Floor and/or ceiling effect was observed in the SF-36 subscales of Physical Functioning, Role–Physical, Social Functioning, and Role–Emotional, which were therefore not included in the evaluation of construct validity (Table 2).

**Table 2:** Floor/ceiling effect of the NOOS, SF-36, and NDI.

PRO instrument	Mean ± SD	Median (min–max)	Floor effect (%)	Ceiling effect (%)
NOOS, score 0–100 (n = 208)				
Mobility	70.49 ± 13.58	70.68 (36.54–100.00)	0.00	1.40
Symptoms	65.51 ± 12.59	65.00 (15.00–96.50)	0.00	0.00
Sleep disturbance	70.45 ± 18.80	68.41 (25.00–100.00)	0.00	7.20
Everyday activity and pain	66.32 ± 13.78	67.22 (31.78–97.72)	0.00	0.00
Participation in everyday life	70.96 ± 15.48	71.86 (28.28–100.00)	0.00	1.00
SF-36, score 0–100 (n = 208)				
Physical functioning	75.65 ± 19.94	80.00 (15.00–100.00)	0.00	16.80
Role–physical	55.85 ± 38.58	50.00 (0.00–100.00)	19.20	33.20
Bodily pain	54.96 ± 20.66	57.50 (0.00–100.00)	0.50	1.90
General health	56.12 ± 19.57	55.00 (10.00–100.00)	0.00	1.90
Vitality	49.37 ± 20.46	50.00 (0.00–100.00)	1.40	0.50
Social functioning	68.77 ± 21.33	75.00 (0.00–100.00)	1.00	15.90
Role–emotional	53.87 ± 40.94	66.60 (0.00–100.00)	26.40	36.50
Mental health	60.42 ± 18.33	64.00 (8.00–96.00)	0.00	1.00
NDI, score 0–50 (n = 208)	75.65 ± 19.94	80.00 (15.00–100.00)	0.50	0.00

### 3.2. Internal consistency

As Cronbach’s alpha values were higher than 0.80 for all NOOS subscales, it was considered that all subscales have sufficient internal consistency (Table 3).

**Table 3:** Test–retest reliability and internal consistency values of the NOOS.

NOOS,0-100 (n = 71)	Baseline mean ± SD	Retest mean ± SD	Difference mean ± SD	ICC (95% CI)	SEM	MDC_ind_	MDC_group_	Cronbach’s α
Mobility	68.92 ± 13.00	70.50 ± 12.31	1.58 ± 7.39	0.819 (0.725–0.883)	5.16	12.16	1.44	0.916
Symptoms	64.15 ± 13.78	67.23 ± 12.21	3.08 ± 9.74	0.721 (0.583–0.817)	6.84	17.12	2.03	0.847
Sleep disturbance	69.66 ± 17.37	70.13 ± 16.44	0.47 ± 10.77	0.803 (0.70–0.872)	5.62	18.50	2.20	0.855
Everyday activity and pain	65.90 ± 13.49	68.29 ± 15.06	2.39 ± 9.27	0.789 (0.680–0.863)	4.85	15.12	1.79	0.889
Participation in everyday life	70.10 ± 15.81	70.52 ± 16.38	0.42 ± 8.83	0.844 (0.761–0.900)	4.68	14.22	1.69	0.915

### 3.3. Test–retest reliability and measurement error

Descriptors of test, retest, and differences for all NOOS subscales among the 71 retested patients, ICC, SEM, MDCind, MDCgroup, and Cronbach’s alpha values are presented in Table 3. In general, test–retest reliability was sufficient for all NOOS subscales of which ICC values were between 0.721 and 0.844. While ICC values calculated for NOOS subscales of mobility, sleep disturbance, and participation in everyday life had good reliability, ICC values calculated for symptoms and everyday activity and pain were found to have acceptable reliability [24]. Standard errors of measurement for NOOS subscales were between 4.68 and 6.84 with participation in everyday life having the lowest value and symptoms having the highest value. MDCind varied between 12.16 and 18.50 with sleep disturbance being the subscale with the highest value. Regarding the assumption that individuals would experience an actual change, the highest MDCind value for sleep disturbance should be at least 18 points [24]. As for the MDCgroup values, the subscale with the lowest value was found to be mobility and the subscale with the highest value was found to be sleep disturbance (Table 3). When taking the highest value as a basis, the change of 2.20 in the sleep disturbance subscale might refer to an actual change only for the group [24].

### 3.4. Construct validity

Eight prespecified hypotheses were tested to confirm construct validity [24]. Since all of the hypotheses were met, it was considered that construct validity is present (Table 4) [19]. 

**Table 4:** Construct validity test: comparison of NOOS subscales, SF-36 subscales, and NDI items

Hypotheses	Correlation coefficients	Confirmed hypothesis
The NOOS subscale “everyday activity and pain” and SF-36 subscale “bodily pain” have a strong correlation (≥0.50)	0.656	
The correlation coefficient between NOOS subscale “everyday activity and pain” and SF-36 subscale “bodily pain” should be higher than the correlation coefficient between the NOOS subscale “everyday activity and pain” and SF-36 subscale “physical functioning”	0.537	
The NOOS subscale “mobility” and SF-36 subscale “general health” have a weak correlation (≤0.29)	0.282	
The NOOS subscale “sleep disturbance” and SF-36 subscale “general health” have a weak correlation (≤0.29)	0.290	
The NOOS subscale “symptoms” and NDI item number 5 (headaches) have at least moderate correlation (≥0.30)	–0.645	
The NOOS subscale “symptoms” and NDI item number 6 (concentration) have at least moderate correlation (≥0.30)	–0.372	
The NOOS subscale “participation in everyday life” and NDI item number 7 (work) have at least moderate correlation (≥0.30)	–0.479	
The NOOS subscale “participation in everyday life” and NDI item number 10 (recreation) have at least moderate correlation (≥0.30)	–0.559	

### 3.5. Factor analysis

Results of five-factor CFA using 208 patients’ responses to the questionnaire revealed that all items were loaded with predetermined factors with over 0.40 factor loads (Table 5). A relationship of 0.80 was found between the factors and it showed that the items creating the five factors were appropriate factors to measure neck pain in patients. The goodness of fit statistic CFI was found to be 0.907, the TLI value was found to be 0.932, and the RMSEA value was found to be 0.057, showing that the five-dimensional structure determined for the NOOS questionnaire was also valid for patients with neck pain in Turkey.

**Table 5:** Confirmatory factor analyses with rotation–pattern matrix (n = 208)

Factor 1 (mobility)	Factor 2 (symptoms)	Factor 3 (sleep disturbance)	Factor 4 (everyday activity and pain)	Factor 5 (participating in everyday life)
M1. 0.675	SY1. 0.697	SL1. 0.878	A1. 0.734	PT1. 0.834
M2. 0.811	SY2. 0.749	SL2. 0.826	A2. 0.683	PT2. 0.798
M3. 0.677	SY3. 0.670	SL3. 0.836	A3. 0.785	PT3. 0.704
M4. 0.794	SY4. 0.756	SL4. 0.875	A4. 0.722	PT4. 0.835
M5. 0.761	SY5. 0.602		A5. 0.784	PT5. 0.790
M6. 0.736			A6. 0.815	PT6. 0.815
M7. 0.705			A7. 0.742	PT7. 0.897
			A8. 0.748	PT8. 0.849
				PT9. 0.763
				PT10. 0.694

## 4. Discussion

We aimed to translate the NOOS into Turkish and assess the questionnaire’s validity and reliability. The Turkish version of the NOOS was in general very good with no disturbing questions and few confusing items. Since the NOOS is not yet available in any other translation, we can only compare the results with the original version. The psychometric properties of the Turkish NOOS were similar to those of the original NOOS in general. Cronbach’s alpha coefficients were calculated to be 0.916 for mobility, 0.847 for symptoms, 0.855 for sleep disturbance, 0.889 for everyday activity and pain, and 0.915 for participation in everyday life. These values indicate that the questionnaire’s internal consistency is at a sufficient level [19,27]. In the original version, Cronbach’s alpha coefficients were calculated to be 0.85 for mobility, 0.77 for symptoms, 0.86 for sleep disturbance, 0.92 for everyday activity and pain, and 0.92 for participation in everyday life. No internal consistency analysis was performed for the NDI. Like the original version of the NOOS, internal consistency of the Turkish version was found to be high.

The ICC method was used for the test–retest analysis in this study. ICC results of the NOOS-Tr varied between 0.721 and 0.844, and time-dependent invariance of the questionnaire is good. In the original version, the test–retest reliability was found between 0.88 and 0.95. The NDI’s test–retest reliability was found as 0.979. There might be a few reasons why the NOOS-Tr had lower test–retest results than the original version and the NDI. The reliability of the Turkish version of the NDI was calculated with 88 patients suffering from chronic neck pain. That number of samples was lower than the number of patients in this study. Moreover, all other language versions of the NDI were examined in two separate systematic compilation studies. As stated in the compilations, reliability values of the studies with the highest quality among the NDI versions were found to be low. Cleland et al. reported that reliability values of two studies with the best quality, which were conducted with large sample sizes and more extensive statistical analyses, were 0.50 and 0.68 [28,29]. In another systematic analysis, it was stated that other language versions of the NDI grouped the patients as acute and chronic and these studies reported that patients suffering from acute pain had lower reliability values [30–34]. Only the patients who were suffering from chronic neck pain for more than 3 months were included in the NDI study. Considering all these findings, the fact that about half of the participant patients in this study had acute neck pain and the high number of the participants might be the reasons why its reliability value was found to be lower than that of the NDI. Almost all of the patients included in the original study of the NOOS had chronic neck pain. About half of the patients in this study had acute neck pain while the other half had chronic neck pain. The patients with acute neck pain might have caused the NOOS-Tr reliability values to be lower than those of the original version. This study also provided values for u2959 and MDC for this test, which can be useful for future studies and clinical practice. The SEM for the NOOS subscales showed “participation in everyday life” having the lowest value and “symptoms” having the highest value. In the original version, the SEMs were lowest for the “everyday activity and pain” subscale and highest for the “symptoms” subscale. MDC values were found to be highest in both studies with 18 points for the sleep disturbance subscale. The score of 18 points for the sleep disturbance subscale was maintained in the 90% confidence interval, which means that it is not a measurement error due to random variation and that there will be true changes in each individual in the future. No floor or ceiling effects were observed. In accordance with the original English version of the NOOS, reliability was satisfactory. 

Construct validity was evaluated in accordance with the COSMIN recommendations. All predefined hypotheses support the construct validity. We evaluated the correlation between the specific NDI parameters and related NOOS subscales when determining the construct validity of our study. The correlations were similar to those of the original version. The mobility and sleep disturbance subscales of the scale and general health subscale of SF-36 were in low correlation in both studies. There was a moderate correlation between the related subscales of the NDI and NOOS in both studies. Since the hypothesis available in the studies of the new versions was tested, it is stated that a comparison between the factor structures of the adapted scale and the original version is required [35]. The NOOS-Tr was found to have five factors, like its original version. The results show parallelism with the results achieved in this study. Since the NDI is a one-factor questionnaire, no factor analysis was performed in the version study. In a study, the one-factor structure of the NDI was criticized in terms of dimensionality and found insufficient [36]. A single summarized score may be difficult as in the NDI because it is not clear how rates of scores will be attributed to each of multiple structures. In the NOOS-Tr, expression of restrictions causing or stemming from neck pain through quantitative data enables them to be interpreted separately and more objectively. These factors reinforce the reliability of the Turkish version of the questionnaire. In the systematic compilation study published by Wiitavaara et al. on the quality of questionnaires that evaluate neck pain patients, the criterion validity was found insufficient for the NDI and sufficient for the NOOS [37]. All these results show that the NOOS-Tr questionnaire can evaluate neck pain patients multidimensionally. 

In conclusion, the NOOS-Tr was determined to be a valid and reliable questionnaire for evaluating neck pain patients. With the Turkish translation of the NOOS and proving its Turkish validity and reliability, it will be possible for Turkish researchers and clinicians to evaluate Turkish patients who have neck pain complaints multidimensionally. Those who want to perform multidimensional evaluation of patients with neck pain may utilize the NOOS-Tr.
